# Lack of association of the iNOS gene polymorphism with risk of cancer: a systematic review and Meta-Analysis

**DOI:** 10.1038/srep09889

**Published:** 2015-09-22

**Authors:** Jinghua Jiao, Jingyang Wu, Desheng Huang, Lei Liu

**Affiliations:** 1 Department of Anesthesiology, Central Hospital, Shenyang Medical College. Shenyang, 110024, China; 2 Department of Ophthalmology, The First Affiliated Hospital, China Medical University. Shenyang, 110001, China; 3 Department of Epidemiology, School of Public Health, China Medical University, Shenyang, 110001, China

## Abstract

In order to investigate the association between the iNOS gene polymorphisms and susceptibility to cancer, a search of English papers was done using Pubmed, the Cochrane Library, Embase, ISI Web of Science, Google (scholar) database, and all Chinese reports were conducted using CBMDisc, Chongqing VIP database, and CNKI database. A total of eight studies were included in this meta-analysis including 1,920 cases and 2,373 controls. The results indicated that the polymorphisms in iNOS gene (C150T(Ser^608^ Leu) polymorphism and polymorphic (CCTTT)n repeats) had no association with cancer risk for all genetic models. This meta-analysis suggested that the polymorphisms in the iNOS gene were not associated with cancer risk.

It has been widely accepted that cancer was one of the important causes of dead all over the world, characterized by angiogenesis and inflamation^1^. To date, the etiology of cancer remains unknown and disease-modifying medical treatments are limited. Nevertheless, since the involvement of cytokines in cancer was hypothesized^2^, there were a lot of candidate genes approach in a case-control study of single nucleotide polymorphisms (SNPs) including inducible nitric oxide synthase (iNOS)^3^,^4^,^5^,^6^,^7^,^8^,^9^,^10^.

According to previous records, iNOS has played an important role in angiogenesis, stimulation of proliferation, and inhibition of apoptosis^11^. Previous research indicated that the iNOS gene might be involved in the development of cancer through disrupting carcinogenesis^12^. The chromosome allocation of iNOS is 17q11.2, which has a genomic size of 48 kb and encodes a protein of 131 kDa^13^. There were some known iNOS gene polymorphisms such as C150T (Ser^608^ Leu) polymorphisms and polymorphic (CCTTT)n repeats.

Thus far, previous studies concerning association between iNOS gene polymorphism and risk of cancer are limited and rather conflicting^3^,^4^,^5^,^6^,^7^,^8^,^9^,^10^. Because lack the evidence to provide a reliable conclusion in a single study, we conducted a meta-analysis on these eligible studies, to evaluate the strength relationship between the iNOS gene polymorphism and risk for cancer, which would have a much greater possibility of reaching reasonably strong conclusions.

## Results

### Study inclusion and characteristics

**Figure 1:**
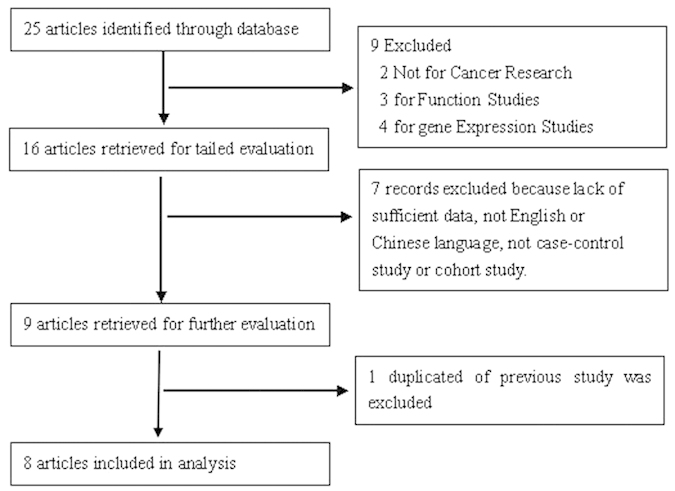
Flow chart demonstrating those studies that were processed for inclusion in the meta-analysis.

The process of the search was shown in Fig. 1. After a literature search, eight studies^3^,^4^,^5^,^6^,^7^,^8^,^9^,^10^(six studies^3^,^4^,^6^,^7^,^8^,^9^ with 1512 cases and 1942 controls for C150T (Ser^608^ Leu) polymorphism and two studies^5^,^10^ with 408 cases and 431 controls for polymorphic (CCTTT)n repeats) were included in this meta-analysis. The identified studies and their main characteristics were shown in Table 1 and Table 2. Genotype distribution of any polymorphism did not differ from HWE within control groups.

### Quantitative data synthesis

**Figure 2:**
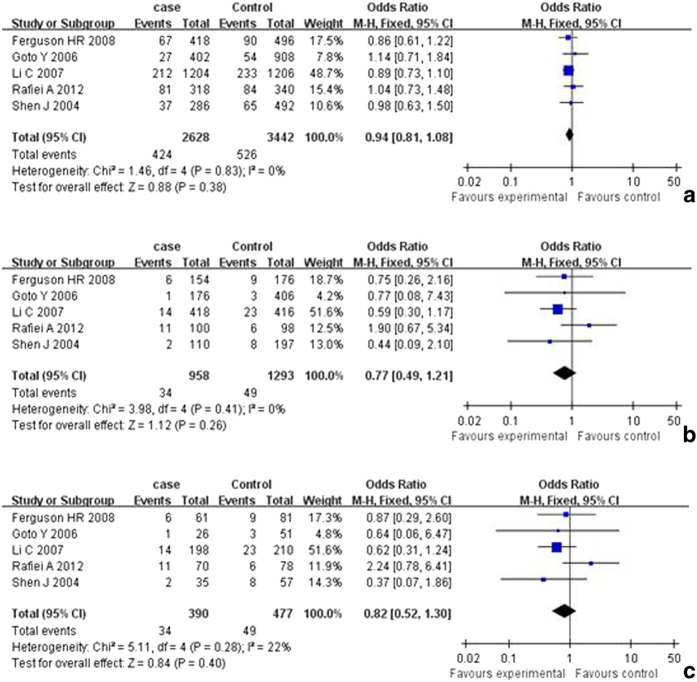
**a**. Forest plot of the association between cancer and the C150T(Ser^608^ Leu) mutation (T vs C). **b**. Forest plot of the association between cancer and the C150T(Ser^608^ Leu) mutation (TT vs CC). **c**. Forest plot of the association between cancer and the C150T(Ser^608^ Leu) mutation (TT vs CT).

**Figure 3:**
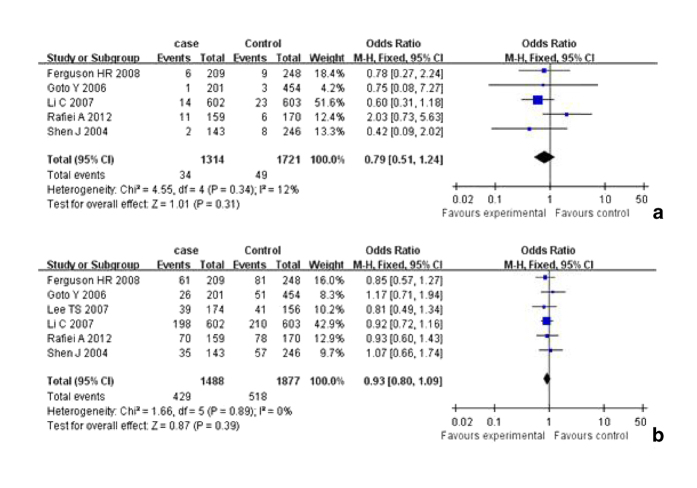
**a**. Forest plot of the association between cancer and the C150T(Ser^608^ Leu) mutation (TT vs CT+CC). **b**. Forest plot of the association between cancer and the C150T(Ser^608^ Leu) mutation (CT+TT vs CC).

**Figure 4:**
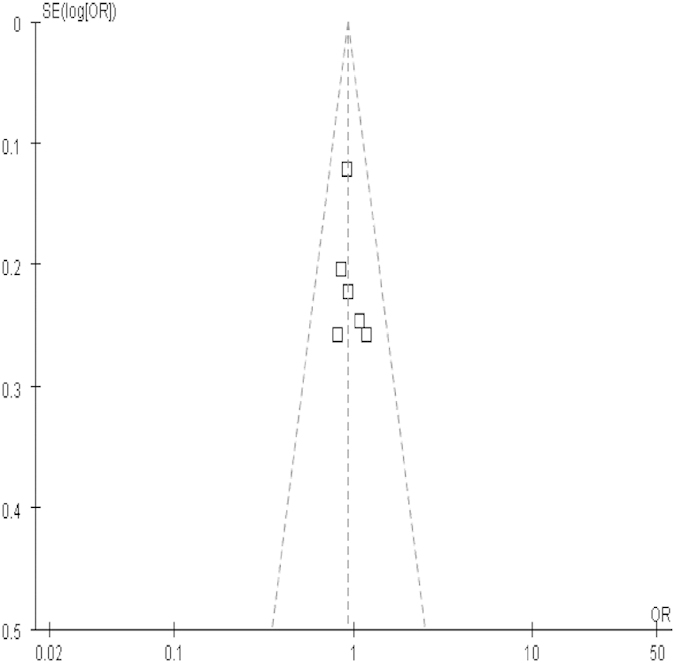
Funnel plot of studies conducted on the association between C150T(Ser^608^ Leu) mutation and cancer risk.

The results of analysis for different polymorphisms by Revman software was shown in Fig. 2 and Fig. 3. As shown in Table 3, the results indicated that there was no association between polymorphisms in the iNOS gene including C150T (Ser^608^ Leu) polymorphisms and polymorphic (CCTTT)n repeats and risk of cancer. In subgroup analysis for C150T (Ser^608^ Leu) polymorphism by ethnicity (OR = 1.04, 95% CI = 0.82-1.32 for T vs. C; OR = 1.10, 95% CI = 0.52-2.33 for TT vs. CC; OR = 1.13, 95% CI = 0.53-2.41 for TT vs. CT; OR = 1.14, 95% CI = 0.54-2.38 for recessive model; OR = 0.98; 95% CI = 0.77-1.25 for dominant model) among Asians. There was no significant publication bias according to funnel plot (Fig. 4) and Egger’s test (p = 0.65).

### Sensitivity Analysis

In order to examine the influence of the individual data set to the pooled ORs, every single study was deleted each time. Sensitivity analysis indicated our results were statistically robust (data not shown).

## Discussion

As a production of nitric oxide (NO), iNOS is produced during inflammation by macrophages^14^. Expression of iNOS in response to cytokines is one of the important sections for inflammatory reaction and relates to angiogenesis, suggesting its potential role in the process of carcinogens^15^. The polymorphism (C150T) in exon 16 of iNOS gene results in an amino acid substitute, Ser^608^ Leu. The iNOS Ser^608^ Leu allele (C > T polymorphism) leads an amino acid alteration in a regulatory domain of the enzyme^16^. Furthermore, a polymorphic pentanucleotide (CCTTT)n repeat probably 2.5 kilobase substream the transcription initiation site has been clarified to affect iNOS expression^17^. In our meta-analysis, the associations between these two polymorphisms in iNOS gene and risk of cancer were studied.

Except detection the expression of iNOS *in vivo* or *in vitro* with tumor^18^,^19^, there were some other researches in cancer patients that susceptibility to carcinogenesis may be correlated with the existence of particular alleles at the iNOS locus. However, the results are inconsistent and inconclusive due to limited sample size and different study methods. To the best of our knowledge, this is the first meta-analysis to evaluate iNOS gene polymorphism in development and growth of cancer. In order to acquire a more reliable and comprehensive evidence on both variants, we conducted this meta-analysis to evaluate the association between the polymorphism in iNOS gene and risk of cancer on the basis of data from eight studies. The results indicated that there was no significant association for iNOS gene polymorphism and risk of cancer. In addition, another iNOS gene (iNOS974) was studied in the relationship with cancer. However, there was no significant difference between iNOS974 polymorphism and cancer risk^9^.

Although we have not found a significant association between iNOS gene polymorphisms and cancer risk, some studies revealed that the risk of cancer is increasing among smoking or drinking individuals with polymorphism in iNOS gene^5^,^6^. In addition, studies also reported that iNOS gene polymorphism was associated with the risk of *H pylori*-related gastric cancer^3^,^8^. We could provide a hypothesis that there was an association between iNOS gene and risk of cancer combined with risk factors including lifestyle and *H pylori* infection. As the reason for heterogeneity (including different study methods and outcomes) among studies, we could not use meta-analysis to analyze the relationship between iNOS genes combined with those risk factors and cancer.

This meta-analysis has pooled the available data from the eligible studies, which has significantly increased the statistical power. Nevertheless, the results of the present meta-analysis should also be explained within the context of its limitations. First, cancer is a multi-factorial illness from complex interactions between environmental exposure and genetic factors. In this meta-analysis, we had insufficient data to conduct an evaluation of such interactions for the role of iNOS polymorphisms and factors in cancer development. Second, the number of current studies is relative limited. Thus, investigations involving large number of different races are necessary for a more reliable assessment on their associations. Third, our meta-analysis is based on unadjusted estimate because lacking of sufficient data. Forth, although we have made our best efforts to avoid all potential publications, it is likely that some are missed or displayed erroneously. Furthermore, we did not include studies published in language other than English or Chinese.

In conclusion, this meta-analysis revealed that the polymorphisms in iNOS gene could not be regarded as a strong genetic risk factor for cancer but it might be association with cancer combined with additional risk factors. In addition, this result should be interpreted cautiously. In order to better understand the potential etiology for cancer in human, large well-designed studies in the susceptibility of cancer evidence are needed to perform in future. It also will be necessary to combine genetic factors and other environmental risk factors.

## Methods

### Selection of eligible studies

Studies on the associations between iNOS gene polymorphisms and cancer were scrutinized by two reviewers (L.L. and J.H.J.) independently. We searched Pubmed, Embase, the Cochrane Library, ISI Web of Science, Google (scholar) database, Chinese Biological Medicine Disc (CBMDisc), China National Knowledge Infrastructure (CNKI), and Chongqing VIP database (Last search was updated on December 10, 2014) using the terms “inducible nitric oxide synthase or iNOS”, “cancer or tumor or carcinoma” and “polymorphism, variant or mutation”. Reference lists were checked and additional literatures were contacted by researchers. Authors of publications were contacted when results were unclear or when sufficient data were not reported. The search was performed without restriction on language, but we only included articles written in English or Chinese.

### Selection Criteria

Studies were included if they fulfilled all of the following entry criteria: (1) it must be a case-control or cohort study design; (2) there were sufficient data for iNOS gene mutations with risk of cancer; (3) the genotype distribution in the controls of all studies should be in agreement with Hardy-Weinberg equilibrium (HWE); and (4) in the case of multiple publications from the same study group, the most complete and recent results were used.

### Exclusion criteria

The exclusion criteria were defined as: (1) abstracts, reviews and studies on animal; (2) useless data reported, genotype number or frequency was not included; and (3) genotype distribution in the control population not consistent with HWE.

### Data extraction

After studies selection, two investigators independently extracted data from each study with a standard form and entered into a database. When discrepancies were appeared, all investigators were recruited to evaluate the data. The following information was collected: First author, publication year, location, ethnicity, characteristics, sample sizes of patients and controls, genotype numbers.

The review and analysis were guided to conduct by the PRISMA statement for preferred reporting of systematic reviews and meta-analysis^20^.

### Statistical analysis

Odds ratios (ORs) with 95% confidence intervals (CIs) for genotypes and alleles were used to assess the strength of association between iNOS gene polymorphisms and risk of cancer. The ORs were conducted for the allele contrast, codominant model, recessive model, and dominant model, respectively. Heterogeneity was examined with *I*^*2*^ statistic interpreted as the proportion of total variation contributed by between-study variation. If there was significant heterogeneity, the random effects model would be used to analyze the pooled ORs^21^,^22^. Otherwise, the pooled ORs were analyzed by the fixed effects model^23^. The potential publication bias was assessed with funnel plot and Egger’s test. An asymmetric plot suggests a possible publication bias and the P value of Egger’s test being considered representative of significant publication bias if it was less than 0.05^24^. All statistical tests were performed by RevMan software (version 5.0, Review Manager, Copenhagen: The Nordic Cochrane Centre, The Cochrane Collaboration, 2010) and Comprehensive Meta-Analysis software version 2.0 (Biostat, Englewood Cliffs, I.N.J., USA). *P* value less than 0.05 for any test was considered to be statistically significant.

## Additional Information

**How to cite this article**: Jiao, J. *et al*. Lack of association of the iNOS gene polymorphism with risk of cancer: a systematic review and Meta-Analysis. *Sci. Rep.* doi: 10.1038/srep09889 (2015).

## Figures and Tables

**Table 1:** Characteristics of the Included Studies for Meta-analysis.

First Author	PublicationYear	Location	Ethnicity	Histology	GenotypingMethod	InosPolymorphism	Cases(n)	Controls(n)	Cconflicts
Rafiei A^3^	2012	Iran	Asian	Gastric cancer	PCR	C150T(Ser^608^ Leu)	159	170	Significant association
Ferguson HR^4^	2008	Ireland	Caucasian	Esophageal adenocarcinoma	TaqMan	C150T(Ser^608^ Leu)	209	248	No significant association
Shen CH^5^	2007	Taiwan	Asian	Bladder carcinoma	PCR	(CCTTT)n	250	250	Significant association
Lee TS^7^	2007	Korea	Asian	Cervical cancer	PCR	C150T(Ser^608^ Leu)	176	172	No significant association
Li C^9^	2007	USA	Caucasian	Cutaneous melanoma	PCR	C150T(Ser^608^ Leu)	602	603	No significant association
Goto Y^8^	2006	Japan	Asian	Gastric cancer	PCR-RFLP	C150T	201	454	Significant association
Tatemichi M^10^	2005	Japan	Asian	Gastric cancer	PCR	(CCTTT)n	158	181	Significant association
Shen J^6^	2004	China	Asian	Gastric cancer	PCR	C150T(Ser^608^ Leu)	165	295	Significant association

PCR: polymerase chain reaction

**Table 2:** Distributions of the inducible nitric oxide synthase Genotype and Allele among Cases and Controls.

	Distribution of C150T(Ser^608^ Leu)genotypes Case/comtrol(n)				Frequency of C150T(Ser^608^Leu)allelesCase/comtrol(n)			Distribution of (CCTTT)nrepeats *genotypesCase/comtrol(n)				Frequency of (CCTTT)n repeats*alleles Case/comtrol(n)	
first author	CC	CT	TT	HWE forcontrol	C	T	first author	SS	SL	LL	HWE forcontrol	S	L
Ferguson HR^4^	148/167	55/72	6/9	0.72	351/406	67/90	Shen CH^5^	45/56	113/110	87/79	0.14	203/222	287/268
Lee TS^7^	135/115	NA	NA	-	NA	NA	Tatemichi M^10^	60/82	75/78	23/21	0.71	195/242	121/120
Li C^9^	404/393	184/187	14/23	0.89	992/973	212/233	-	-	-	-	-	-	-
Shen J^6^	108/189	33/49	2/8	0.06	249/427	37/65	-	-	-	-	-	-	-
Rafiei A^3^	89/92	59/72	11/6	0.07	237/256	81/84	-	-	-	-	-	-	-
Goto Y^8^	175/403	25/48	1/3	0.24	375/854	27/54	-	-	-	-	-	-	-

NA/-: Not applicable. HWE: Hardy-Weinberg equilibrium. *repeat numbers divided into two groups: S (9-11repeats) and L (12-18repeats).

**Table 3:** Summary ORs and 95% CI of the C150T(Ser^608^ Leu) Polymorphism and the polymorphic (CCTTT)n repeats in the inducible nitric oxide synthase Gene and Cancer Risk.

Statistical models	Genotype/Allele	Numberof Study	OR	95%CI	I^2^%	P	Z	*P* forZ test
C150T(Ser608 Leu)								
Allele Model	T vs. C	5	0.94	0.81-1.08	0	0.83	0.88	0.38
Codominant model	TT vs. CC	5	0.77	0.49-1.21	0	0.41	1.12	0.26
	TT vs. CT	5	0.82	0.52-1.30	22	0.28	0.84	0.4
Recessive model	TT vs. CT+CC	5	0.79	0.51-1.24	12	0.34	1.01	0.31
Dominant model	TT+CT vs. CC	6	0.93	0.80-1.09	0	0.89	0.87	0.39
Subgroup								
C150T(Ser608 Leu) in Asian								
Allele Model	T vs. C	3	1.04	0.82-1.32	0	0.90	0.35	0.72
Codominant model	TT vs. CC	3	1.10	0.52-2.33	0	0.29	0.24	0.81
	TT vs. CT	3	1.13	0.53-2.41	46	0.16	0.31	0.75
Recessive model	TT vs. CT+CC	3	1.14	0.54-2.38	31	0.23	0.34	0.74
Dominant model	TT+CT vs. CC	4	0.98	0.77-1.25	0	0.74	0.14	0.89
(CCTTT)n repeats*								
Allele Model	L vs. S	2	1.20	0.99-1.46	0	0.75	1.83	0.07
Codominant model	LL vs. SS	2	1.41	0.95-2.11	0	0.84	1.69	0.09
	LL vs. SL	2	1.09	0.77-1.54	0	0.88	0.49	0.63
Recessive model	LL vs. SL+SS	2	1.19	0.86-1.65	0	0.76	1.07	0.29
Dominant model	LL+SL vs. SS	2	1.33	0.98-1.82	0	0.93	1.83	0.07

OR: odds ratio; CI: confidence interval. *repeat numbers divided into two groups: S (9-11 repeats) and L (12-18 repeats).
